# Untangling the evolution of Rab G proteins: implications of a comprehensive genomic analysis

**DOI:** 10.1186/1741-7007-10-71

**Published:** 2012-08-08

**Authors:** Tobias H Klöpper, Nickias Kienle, Dirk Fasshauer, Sean Munro

**Affiliations:** 1 MRC Laboratory of Molecular Biology, Hills Road, Cambridge CB2 0QH, UK; 2 Department of Cellular Biology and Morphology, University of Lausanne, Rue du Bugnon 9, 1005 Lausanne, Switzerland

**Keywords:** Organelles, G proteins, humans, last eukaryotic common ancestor

## Abstract

**Background:**

Membrane-bound organelles are a defining feature of eukaryotic cells, and play a central role in most of their fundamental processes. The Rab G proteins are the single largest family of proteins that participate in the traffic between organelles, with 66 Rabs encoded in the human genome. Rabs direct the organelle-specific recruitment of vesicle tethering factors, motor proteins, and regulators of membrane traffic. Each organelle or vesicle class is typically associated with one or more Rab, with the Rabs present in a particular cell reflecting that cell's complement of organelles and trafficking routes.

**Results:**

Through iterative use of hidden Markov models and tree building, we classified Rabs across the eukaryotic kingdom to provide the most comprehensive view of Rab evolution obtained to date. A strikingly large repertoire of at least 20 Rabs appears to have been present in the last eukaryotic common ancestor (LECA), consistent with the 'complexity early' view of eukaryotic evolution. We were able to place these Rabs into six supergroups, giving a deep view into eukaryotic prehistory.

**Conclusions:**

Tracing the fate of the LECA Rabs revealed extensive losses with many extant eukaryotes having fewer Rabs, and none having the full complement. We found that other Rabs have expanded and diversified, including a large expansion at the dawn of metazoans, which could be followed to provide an account of the evolutionary history of all human Rabs. Some Rab changes could be correlated with differences in cellular organization, and the relative lack of variation in other families of membrane-traffic proteins suggests that it is the changes in Rabs that primarily underlies the variation in organelles between species and cell types.

## Background

The secretory and endocytic pathways of eukaryotic cells allow the biosynthesis of lipids and of secreted and membrane proteins to be separated from the barrier function of the plasma membrane. They also allow the remodeling of the cell surface and the uptake of large molecules and even of other organisms. Although some prokaryotes have internal organelles, these lack the complexity seen in eukaryotes and also the trafficking routes that connect the organelles into pathways [1]. However, this complexity and connectivity presents eukaryotic cells with a major organizational challenge, as vesicles and other carriers must select particular cargo from their site of generation, and then move toward, and fuse with, specific target organelles. The specificity of cargo selection is determined by coat proteins and their adaptors, with the recruitment of coats being directed by phosphoinositides or by members of the Arf family of small G proteins [2,3]. However, the movement and arrival of vesicles is directed for the most part by small G proteins of the Rab family, with the soluble N-ethylmaleimide-sensitive-factor attachment protein receptors (SNAREs) then driving membrane fusion [4,5].

Originally identified in yeast, the Rab proteins have now emerged as the largest and most diverse family within the 'landmark' molecules that specify the identity of vesicles and organelles [6-10]. They are typically anchored to the bilayer by a long flexible hypervariable domain that ends in a prenylated C terminus. In the GTP-bound form, Rabs bind effectors, but when the GTP is hydrolyzed through the action of a GTPase activating protein (GAP), they are extracted from the bilayer by a carrier protein called GDP dissociation inhibitor (GDI) [11,12]. Thus the activating exchange factors (GEFs) and GAPs of Rabs act in concert to establish a restricted subcellular distribution for each particular Rab-GTP form, and this GTP form then serves as a landmark for the recruitment of those proteins that need to act in that location. Rab-GTP effectors include linkers to motor proteins and tethering factors that attach vesicles to the correct organelle before fusion.

Although Rabs are members of the Ras superfamily of small G proteins, they share features that allow them to be clustered within a monophyletic branch of this family, with their closest relatives being the Ran family which directs nuclear transport [13-15]. The genomes of all eukaryotes encode multiple members of the Rab family, with humans having 66 and even the simplest eukaryotes having more than 10 Rabs. [16-18]. Studies of Rab conservation have shown that their numbers and presence varies considerably between different phyla, and even between different species within phyla [13,16]. Thus, studies on Rabs have great potential to shed light on the evolution of eukaryotic endomembrane systems, and have already provided useful insights into this issue [17,19,20]. However, the complex history of Rabs, including independent losses, duplications and diversifications, means that providing a full catalogue of Rab diversity and evolutionary history is a major challenge.

In this study, we used a classification and phylogeny-based approach to define subfamilies of the Rab proteins, and then iteratively built and refined hidden Markov models (HMMs) for these subfamilies to use for searching sequence databases. Finally, we built evolutionary trees using maximum likelihood and distance-based methods. This provided the most comprehensive view of Rab evolution obtained to date, and we have established a *Rab *Database web server to make the classifiers and full analysis available. Examining the patterns of Rab evolution demonstrates the striking degree of Rab complexity in the last eukaryotic common ancestor (LECA), and provides insights into the evolution of membranes prior to the LECA. In addition, it is clear that many Rabs have proven dispensable during evolution whereas a small set have been well conserved, or have even expanded in particular lineages, and in some cases these changes can be correlated with changes in cellular properties, or can even be used to predict function.

## Results

### A hidden Markov model-based classification of Rab protein sequences

We started our classification by collecting known Rab protein sequences from 21 widely diverse species. After aligning the sequences and removing those of low quality, we obtained a first set of approximately 500 sequences from which we reconstructed a phylogenetic tree and clustered similar sequences (see Methods for details). We extracted a conserved core Rab motif from the constructed alignment, partitioned it into sub-alignments according to the different initial sequence groups and constructed an HMM for each one. Finally, we used these models to search genome and expressed sequence tag (EST) databases for further Rab sequences. With the expanded dataset we repeated the analysis and refined the set of specific HMMs. This process was repeated until no further improvement in the classification was achieved. Finally, we supplemented our dataset with data from further species, obtaining more than 7600 different Rab sequences from more than 600 species representing all major eukaryotic phyla. For 384 of these species we found genome projects listed in the Genomes On Line Database (GOLD) [21]. The analysis contained 248 metazoans (of which 131 were included in GOLD: 37 genomes were complete, 3 were draft, and 91 were incomplete), 166 fungi (25, 4, and 90 respectively), 81 plants (16, 1, and 30), 19 apicomplexans (8, 0, and 7), 20 heterokonts (7, 1, and 5), and 11 kinetoplastids (4, 0, and 4).

### Identifying the Rabs of the last eukaryotic common ancestor

**Figure 1:**
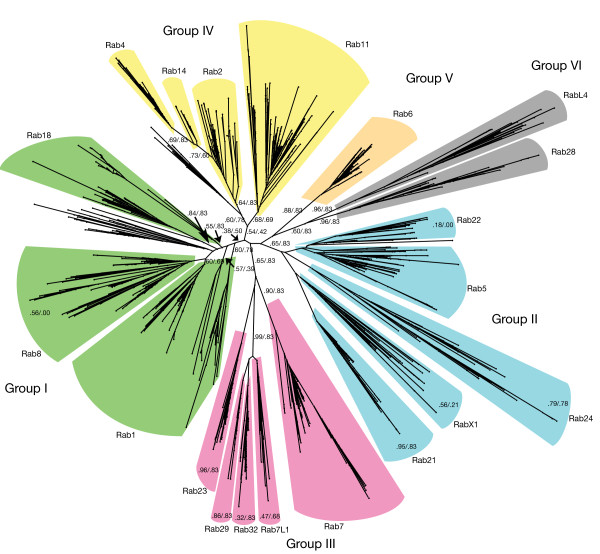
**Evolutionary tree depicting the relationship of the different Rab families that were potentially present in the last eukaryotic common ancestor (LECA)**. The tree was constructed as described in the Methods section; we removed any groups that were difficult to place (Rab19, Rab33, Rab34, Rab44 and Rab45) from the tree. Statistical support is shown for edges that support major Rabs groups, or a LECA Rab group, or show unexpected splits, and also for the colored surroundings associated with the edge, with the first number of the pair being the likelihood-mapping value and the second being the almost unbiased test (AU) value [91]. All supergroups are clearly separated, and most LECA groups showed good statistical support. Exceptions generally showed better support in their corresponding supergroup trees (see Additional file 2). For example, the analysis indicated that Rab22 is a close relative of Rab5. Separating Rab5 and Rab22 was complicated by protozoan duplications of Rab5, which have drifted away from their ancestor and are often placed very close to Rab22.

**Figure 2:**
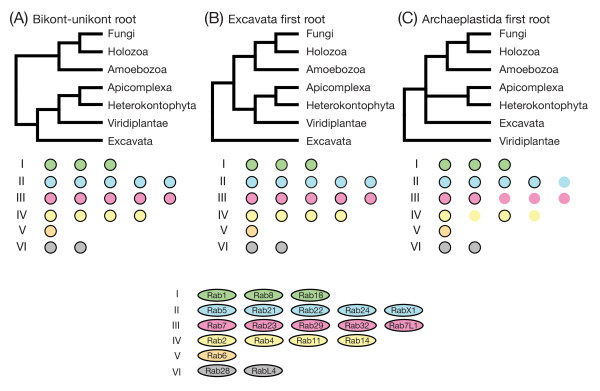
**Changes to the repertoire of Rab proteins in the last eukaryotic common ancestor (LECA) considering different hypotheses of rooting the eukaryotic tree**. **(A-C) **This shows the major hypotheses for rooting the eukaryotic tree, and underneath the six different Rab supergroups with their members (order as shown in the key). Presence of a Rab type in the LECA is indicated by a black outline. Hypotheses A and B both suggest the same 20 Rab types being present in the LECA (see also Figure 3 and Additional file 1). Placing Archaeplastida as an outgroup to all other eukaryotes would decrease the number of Rabs in the LECA to a minimum of 14, although in this model the position of Excavata is uncertain [29].

Our classification analysis identified a set of 20 basic Rab types (Figure 1). Deducing which of these Rab types were present in the LECA was complicated by the fact that the placement of the root in the eukaryotic tree of life is still under debate [22-24]. What is perhaps the most widely accepted hypothesis places the eukaryotic root between the bikonts and unikonts [23,25,26]. Other possible scenarios reported to date include rooting the tree within or close to Excavata [27,28], and studies of rare genomic changes have led to the proposal of a root between Archaeplastidia and all other eukaryotes [29]. We inspected the effect of these different hypotheses on the set of likely LECA Rabs, and found that the presence of the same 20 Rabs in the LECA was supported by two of the three trees (Figure 2). The exception was the tree based on the Archaeplastida outgroup, in which the number of LECA Rabs reduced to 14 of these 20. However, it should be noted that in this model the position of the excavates was uncertain, with the authors stressing the need for 'extreme caution' [29], and if the excavates were placed with the Archaeplastida, then the number of LECA Rabs would again be 20.

**Figure 3:**
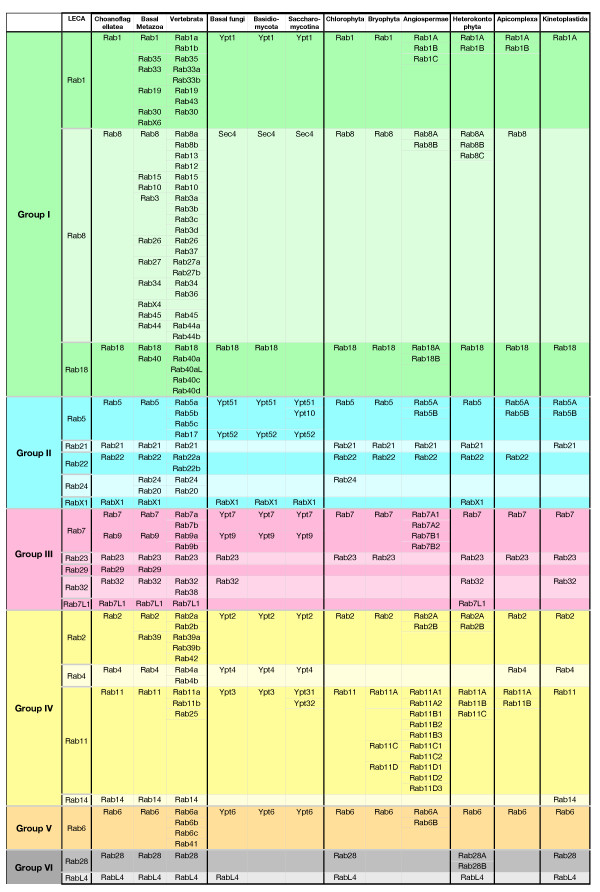
**Last eukaryotic common ancestor (LECA) Rabs and their evolution in different phyla**. In this overview, we show the changes of LECA Rabs in a number of different phyla. The most elaborate changes to the repertoire are displayed. For example, the transition to metazoan multicellularity gave rise to a large number of new, mainly secretory, Rabs. This can be seen by the change from choanoflagellates (*Monosiga brevicollis*) to metazoans. Similarly, there was a loss of LECA Rabs in fungi; basal fungi still possess a large number of these Rabs, but Basidiomycota have already lost three and the Saccharomycotina another one. Early in their evolution, plants lost a number of LECA Rabs, but then expanded the remaining Rabs into families. Particularly in plants there was a number of Rabs that we could only locate in one species: Rab24 was found only in the algae *Coccomyxa*, Rab28 was found only in the algae *Micromonas*, and for angiosperms we could identify a Rab21 only in *Oryza sativa*. It seems likely that these groups will get better support once more genome sequences become available.

It is also formally possible that Rabs have moved between kingdoms by horizontal gene transfer, but the trees for the 20 Rabs generally fit well with species evolution. In addition, horizontal gene transfer after endosymbiosis would probably have involved a non-unikont species as the donor and so would not have increased the number of Rab families in the bikonts. If one divides eukaryotic phyla into the four proposed supergroups (Unikonta, Excavata, Archaeplastida and SAR+CCTH [28]), then of the 20 putative LECA Rabs, 19 are present in at least three (Figure 3 and Additional file 1). The exception was Rab29, whose only occurrences outside of unikonts were found in *Naegleria gruberi *(an excavate) and *Thecamonas trahens *(a member of the phylum Apusozoa, whose relationship to other phyla is unclear) [23,30]. Thus, Rab29 seems to be the only equivocal Rab in our proposed set of 20 LECA Rabs. Given these observations and caveats, we have based our further discussion on the cautious assumption that all 20 basic Rab types were in the LECA.

For each of these 20 basic types we constructed HMMs. However, this basic set has diversified over time in all major phyla, and especially in metazoans. For the majority of these diversifications, it is clear from which LECA Rab they descended; however, we found a number of lineage-specific Rab types that have diverged substantially from their ancestors, making them difficult to place with one of the LECA Rabs. This problem is especially present in protozoan lineages for which only few species have been sequenced to date. For a few metazoan duplications, we used an approach based on sequence similarity to identify their ancestral Rab type (see Methods for details). To better distinguish basic Rabs from those that developed later, we supplemented the generated HMMs with 15 further models that recognize metazoan-specific types, and especially those that were difficult to place. Other eukaryotic lineages with extensive genome sequence coverage, such as fungi and plants, do not possess such diversified Rab sets, and so for these cases we did not need to generate further specific HMMs.

All our models showed at least 95% sensitivity and positive predictive value (see Additional file 2). Thus, the structure of the Rab family tree developed from our analysis appears to be robust, allowing considerable confidence in its implications. We have implemented a web interface called the *Rab *Database which provides access the collected information and allows searches with new proteins against our HMM-based classifiers (http://bioinformatics.mpibpc.mpg.de/rab/).

The history of Rab evolution shows some striking features, including an unexpectedly large number of different Rabs present in the LECA. These twenty LECA Rabs can be arranged into six supergroups, which has implications for the evolution of the membrane system prior to the LECA. During the subsequent divergence of eukaryotes many of the LECA Rabs have been lost in particular lineages, whereas other families have expanded, and in some cases it is possible to correlate this with loss or gain of particular cellular processes. These points are discussed in detail below.

### The last eukaryotic common ancestor had a large repertoire of Rabs

Our analysis identifies a set of 20 Rab proteins that are likely to have been present in the LECA based on the arguments above (Figure 1, Figure 3). Of these proteins, Rab1, Rab2, Rab4, Rab5, Rab6, Rab7, Rab8, Rab11, Rab18, Rab21, Rab23, and Rab28 are known likely candidates, and Rab14, Rab32, and RabL4 have just recently been added to this list [16]. To these proteins we can now add Rab22, Rab24, Rab29, RabX1, and Rab7L1. This total of 20 is larger than that found in many extant eukaryotic phyla, such as plants and fungi. This indicates that the endomembrane system of the LECA was relatively complex, consistent with studies of proteins involved in other cellular processes such as the cytoskeleton, all of which have indicated that the LECA was a particularly complex and sophisticated cell [24,31].

It is well established that the LECA must have had a Golgi apparatus, and the capacity for both endocytosis and phagocytosis [32]. This would be consistent with the roles that many of these Rabs have been reported to play in extant eukaryotes. Thus Rab1, Rab2, and Rab6 are on the Golgi, and Rab8 acts in Golgi to plasma membrane traffic, while Rabs 4, 5, 7, 11, 14, 21, and 22 are in the endosomal system, and are thus likely to have acted in endocytic and phagocytic processes of the LECA cell. These processes will have allowed uptake of food sources, as well of recycling of components to the cell surface, and possibly the fusion of a contractile vacuole to expel water [33,34]. Likewise, RabL4 (also known as intraflagellar transport (IFT)27) and Rab23 are known to be involved in cilia/flagella formation or function in extant eukaryotes, consistent with other proteins specific to these structures being found in all eukaryotic kingdoms and thus present in the LECA [35].

Of the remaining seven LECA Rabs, Rab32 and its paralogs in extant eukaryotes (for example, Rab38) are well established to be involved in forming lysosome-related organelles (LROs) such as melanosomes, platelet dense granules and alveolar lamellar bodies [36,37]. Rab29 and Rab7L1 are distantly related (see below), and the latter has been proposed to also have a role in granules derived from endosomal system [38]. It can only be speculated as to how the LECA may have used LROs, but obvious possibilities include pigment granules to block sunlight, secretory granules to combat competitors, or granules that fused with phagosomes to aid killing of phagocytosed bacteria in the manner of neutrophils [39]. Of the few non-metazoans that have conserved this Rab, phytophthora contain a large number of granules in their motile zoospores, which are released during encystation to rapidly reform the cell wall, concomitant with a loss of motility [40].

This leaves four Rabs (Rab18, Rab24, Rab28 and RabX1) whose role even in extant eukaryotes is unclear, although their ancient origins suggest that they may have more fundamental roles than previously thought. Of these, Rab18 is the best characterized, with several reports suggesting a role on the endoplasmic reticulum (ER), either in lipid droplet formation or in Golgi to ER traffic [41,42]. However, despite Rab18 being well conserved in many eukaryotic phyla, human patients lacking Rab18 do not show obvious defects in lipid storage or general secretion, and so the role of Rab18 remains unclear [43]. However, it is possible that Rab40 may have a partially redundant role with Rab18, as in humans it seems to have expanded from Rab18 during metazoan evolution (see below). Of the remaining three LECA Rabs, Rab24 has been linked to autophagy, but cannot be obligatory for this process as it is conserved in only a very few non-metazoan phyla, and Rab28 has been linked to endosome function in trypanosomes, but little is known of its role in metazoans [44,45]. Finally, RabX1 is conserved in only a few sequenced genomes across several phyla, including various invertebrates but not vertebrates. In *Drosophila*, it is expressed primarily in the nervous system, and a P-element insertion next to the gene perturbs development of the peripheral nervous system [46,47].

### Rab expansion during the period of evolution leading to the last eukaryotic common ancestor

Many eukaryotic-specific genes were already present in the LECA as families, which indicates that they must have duplicated and diverged in the period between the earliest eukaryote(s) and the LECA. The Rabs are a particularly extreme case of this, and our analysis allows insights into how this family emerged. During the iterative refinement of our analysis, it became apparent that the twenty LECA Rabs fell into a set of six larger supergroups, suggesting a primitive pre-LECA eukaryote with just six Rabs. The tree (Figure 1) shows the relationship between the different LECA Rabs, and provides statistical support for this observation. Interestingly, each of these six supergroups comprised Rabs that are mostly associated with one particular process, consistent with diversification from a simpler system in which there was a single Rab for each of the following: secretion (group I), early endosomes (group II), late endosomes (group III), recycling from endosomes to the surface (group IV), recycling from endosomes to Golgi (group V), and traffic associated with cilia/flagella (group VI).

### Rab family evolution during the diversification of the eukaryotes

**Figure 4:**
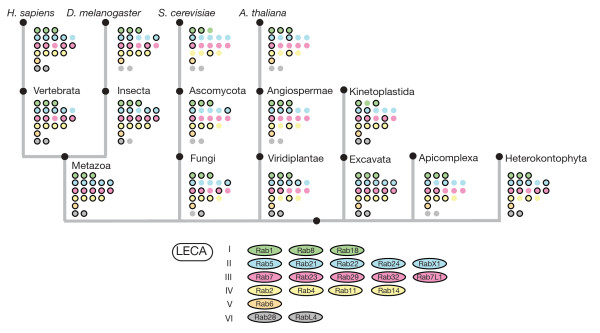
**Overview of how Rab proteins were lost during the expansion of eukaryotes**. The losses of the putative last eukaryotic common ancestor (LECA) Rabs are indicated at each evolutionary step, as depicted in the key, with loss being indicated by loss of the black outline. The tree reflects available genome sequences rather than the full diversity of eukaroytic lineages. For the phyla with sufficient sequences available, we depict the losses during the evolution of a specific model organism. The extent of lost LECA Rab types varies considerably between different phyla. Whereas metazoans seem not to have lost any, the Apicomplexa have lost nine. Similarly, humans seem to have only lost two LECA Rabs, but *S. cerevisiae *has lost a total of fourteen.

Comparing the LECA Rabs with those present in extant eukaryotic phyla revealed two striking patterns. Firstly, many Rabs have been lost in at least some lineages, despite being conserved in others, with relatively few Rabs seeming to be indispensible (Figure 4). Secondly, some Rab families have expanded greatly in particular kingdoms (Figure 3). In both cases, it was sometimes possible to correlate these genetic changes to changes in cell structure and function. Below we discuss these two aspects of Rab evolution in detail.

### Rab losses during eukaryotic evolution

Although the LECA seems to have had at least 20 Rabs, most of these are clearly not essential for eukaryotic life, as many have been lost in one or more kingdoms (Figure 4). Indeed, of the twenty Rabs present in the LECA, only five seem near-indispensible, in that they are present in almost all well-characterized genomes reported to date. These are Rab1, Rab5, Rab6, Rab7 and Rab11, which correspond precisely to five of the six supergroups present in the LECA, with the remaining supergroup probably being that linked to cilia/flagella. This again suggests that membrane traffic is fundamentally underpinned by just five Rabs. Note that even these 'indispensible' five show some losses in a few very reduced or parasitic eukaryotes such as the microsporidian *Encephalitozoon cuniculi*, and although all five are present in the budding yeast *Saccharomyces cerevisiae*, remarkably three of the five (Rab5, Rab6 and Rab7) are not actually essential for the viability of this yeast. In addition to these core five Rabs, there are three Rabs that are only rarely lost in free living eukaryotes: Rab2 and Rab18 (both lost in budding yeasts) and Rab8 (lost in kinetoplastids). It may be that these three can be lost because the 'indispensible' Rab in their group can sometimes take over their role, and, indeed, it has been shown for *S. cerevisiae *that a single chimeric Rab can perform the roles of both Rab1 and Rab8 [48,49].

Consistent with the LECA having a large Rab repertoire, there is evidence that several eukaryotic kingdoms contain more Rabs than do many of that kingdom's individual species (Figure 4). For instance, basal fungi have already lost seven of the twenty LECA Rabs (Rab14, Rab21, Rab22, Rab24, Rab28, Rab29 and Rab7L1), but it is apparent that further Rabs were lost during the expansion of the fungal kingdom. All non-basal fungi have also lost Rab23, Rab32 and RabL4. In addition, Rab18 has been lost in all Saccharomycotina, while Rab2, Rab4 and RabX1 are still present in *Yarrowia lipolytica *but seem to have been lost in all later Saccharomycotina, including *S. cerevisiae*, leaving the latter with orthologs of only six of the LECA Rabs. For plants, only 14 out of the 20 LECA Rabs are present in the Chlorophyta. Interestingly, all more derived plants have lost three additional LECA members (Rab24, Rab28 and RabL4). In addition, angiosperms have also lost Rab23, and so even these complex multicellular eukaryotes lack almost half of the Rabs present in the LECA.

In some cases, it is possible to correlate loss of particular sets of Rabs with changes in cellular organization. Rabs linked to cilia and flagella (RabL4 (IFT27) and Rab23), have been lost in those organisms that have also lost these structures, in particular most plants and fungi. This loss is often associated with the organism gaining a cell wall, and such a structure would also prevent phagocytosis of large objects. This provides a possible explanation for the loss of Rab14 in these lineages, as this Rab is recruited to phagosomes in both mammals and *Dictyostelium *[33,50]. In other cases, genome compaction may have driven Rab loss, as particularly small Rab repertoires are present in organisms with compacted genomes such as the microalgae *Ostreococcus*, or the budding yeasts, although it is also possible that this is an indirect consequence of a general simplification of the intracellular membrane-traffic systems of these organisms.

### Rab expansions

While many Rabs show a history of extensive independent losses, there have also been many cases in which Rab families have expanded by gene duplication and diversification. Metazoans show expansions in twelve different LECA Rabs, plants in eight, heterokonts in five, apicomplexans in three, fungi in two and kinetoplastids in just one (Figure 3; Figure 4). We discuss these expansions briefly for each of the six supergroups of Rabs.

#### Group I: Rab1, Rab8, Rab18

This supergroup has a particularly complex history of expansions and losses. Rab1 is the 'indispensible' member of the supergroup, and seems to have been duplicated a number of times in metazoans (Rab19, Rab30, Rab33, Rab35, RabX6). The addition of Rab35 seems to predate the rise of metazoans, as we could also identify it in *Capsaspora owczarzaki*, which branched off from the pre-metazoan lineage after fungi but before choanoflagellates. RabX6 appeared in metazoans, but is one of the few Rabs that is lost in vertebrates. Rab1 has duplicated independently in most other phyla, including apicomplexans (Rab1B) and heterokonts (Rab1B) [51]. In angiosperms, there are three different Rab1 proteins, apparently expanded from one in Bryophyta.

Rab8 has probably the most complex history of the LECA Rabs. It has been independently triplicated in heterokonts and, similar to Rab1, it has been duplicated in angiosperms but not in Bryophyta. More strikingly, it has a large set of duplications in metazoans (Rab3, Rab10, Rab15, Rab26, Rab27, Rab34, Rab44, Rab45, RabX4). Rab44 and Rab45 are two of three Rabs that have an additional domain present, with two EF hand motifs present in the N-terminal region of the protein. Many of these Rab families have expanded further in vertebrates, but a few, such as RabX4, have been lost. Although RabX4 is present in insects and not in vertebrates, it is also present in a few genomes of more primitive metazoans (for example, *Amphimedon queenslandica, Nematostella vectensis *and *Strongylocentrotus purpuratus*), indicating that it arose early in metazoan evolution and was widely lost. In addition, Rab8 seems to have undergone another duplication in deuterostomes (Rab12) and two in vertebrates (Rab8b, Rab13).

By contrast, Rab18 seems to have a rather simple evolutionary history, being present in all major clades and only being lost in Saccharomycotina. It shows a duplication in angiosperms and also duplication in bilateria (Rab40), which expanded further in vertebrates (Rab40b, Rab40c) and then in primates (Rab40a, Rab40aL).

#### Group II: Rab5, Rab21, Rab22, Rab24 and RabX1

Rab5 is apparently indispensible, and also has the most complex evolutionary history of this supergroup. It seems to have been duplicated independently in basal fungi after the loss of their flagellum (Ypt52), and also in apicomplexans (Rab5B) and kinetoplastids (Rab5B). In addition, it has quadrupled in vertebrates (Rab5a, Rab5b, Rab5c, Rab17). Similarly, there are independent duplications in Saccharomycotina (Ypt10**) **and in Angiospermae (Rab5B).

Rab21 and Rab22 have no major expansions (Rab22 has a duplication in vertebrates), and have been lost in members of several phyla. Rab24 is rather unusual as it is well conserved in metazoans, where it has also been duplicated (Rab20), but it seems to have been lost in most other species. However, we could detect members of this group in a small but diverse group of species outside of unikonts, indicating that it was present in the LECA (Figure 3; see Additional file 1). The final member of this group, RabX1, is one of the few that seem to have been lost in vertebrates; however, it is well conserved in insects and nematodes, as well being present in heterokonts and fungi (it is present in some Saccharomycotina species, but not in *S. cerevisiae*).

#### Group III: Rab7, Rab23, Rab29, Rab32 and Rab7L1

Rab7 seems to be indispensible, and is also the member of the family that shows the most expansions. There are four Rab7s in angiosperms, and metazoans have a Rab7 relative, Rab9. Previous analysis has dated the duplication that formed Rab9 to the rise of the metazoans [52]. However, we identified a conserved set of fungal proteins that must either be an independent duplication of fungal Rab7 or must belong to the Rab9 subfamily, and hence Rab9 appeared with the opisthokonts. Our phylogenetic analysis indicates that the latter possibility seems to be more likely. In addition, we found this special fungal version of the protein not only in a wide variety of fungi, but also in very basal fungi species, strengthening our view that these sequences are indeed members of the Rab9 family.

Rab23 and Rab32 show extensive patterns of loss, which in the case of Rab23 correlate well to loss of cilia or flagella, consistent with functional work on this protein [35]. Rab23 shows no duplications, whereas Rab32 has been duplicated in vertebrates (Rab38). Rab7L1 and Rab29 have been independently lost in fungi, plants, apicomplexans, and kinetoplastids, but for Rab7L1, we identified a number of related sequences in diverse non-unikont species and in metazoans, including humans (Figure 3 and Additional file 1). As noted above, the assignment of Rab29 as a LECA Rab rather than being unikont-specific is based on Rab29 being in one excavate with a high score to our Rab29 model, with a Rab29-like sequence also being found in the phylogenetically elusive Apusozoa. Interestingly, Rab29 has also been lost in vertebrates. The relationship between Rab7L1 and Rab29 is not completely clear, as several metazoan species seem to have lost one or other group. However, we found sequences from choanoflagellates and from *C. owczarzaki*, corresponding to both groups. We have used the names Rab29 and Rab7L1 for these two groups because they have been used for members of the two groups previously; however this has not been consistent, thus if confirmed by more genomes, adopting new names for these two proteins may help avoid confusion.

#### Group IV: Rab2, Rab4, Rab11 and Rab14

Rab11 seems to be the indispensible member of this supergroup, and is certainly the only Rab from this family that is still present in Saccharomycotina, where it has duplicated (Ypt31/32). Overall, Rab11 presents a unique pattern of duplications. Apicomplexa contain two Rab11 genes, and there has been a large expansion in plants. There are three different Rab11 genes in Bryophyta (Rab11A, Rab11C, Rab11D); these genes expanded to 10 versions in angiosperms and *Arabidopsis thaliana *possesses 26 different Rab11 genes. In heterokonts, we can observe some independent duplications of Rab11 genes, including oomycetes which have seven Rab11 genes.

Rab2 is present in all major eukaryotic phyla and has been independently duplicated in metazoa (Rab39), heterokonts (Rab2B) and angiosperms (Rab2B), but has been lost in all more derived Saccharomycotina species. By contrast, Rab4 and Rab14 have no major duplications (Rab4 has been duplicated in vertebrates) and have both been lost in several phyla.

#### Group V: Rab6

Rab6 seems to be indispensible, but we found no expansions in the genomes examined. apart from one in Angiospermae and, like many other Rabs, an expansion in vertebrates (Rab6b). In the latter case, there was a later expansion to generate Rab41, which is present in primates and dolphins, and a further expansion due to a reactivated retrotransposed pseudogene, which seems to be specific to *Hominidae *(Rab6c, [53]).

#### Group IV: Rab28 and RabL4

Neither of these Rabs is indispensible, with many losses and no expansions apart from a duplication of Rab28 in heterokonts. RabL4 (also known as IFT27) has been clearly linked to the function of flagella and cilia, and its pattern of conservation fits well with the presence of these important but not ubiquitous structures [54]. Interestingly, the phylogenetic profile of Rab28 is rather similar to that of RabL4, suggesting that its function may also be linked to cilia or flagella. RabL4 has no prenylation site, and our analysis gives strong support to separating this group from the other Rabs (Figure 1). This may indicate that these groups are not classic Rab proteins, but would be better placed somewhere between the Ran and Rab supergroups of the Ras family.

### What has driven the expansion of particular Rab families?

**Figure 5:**
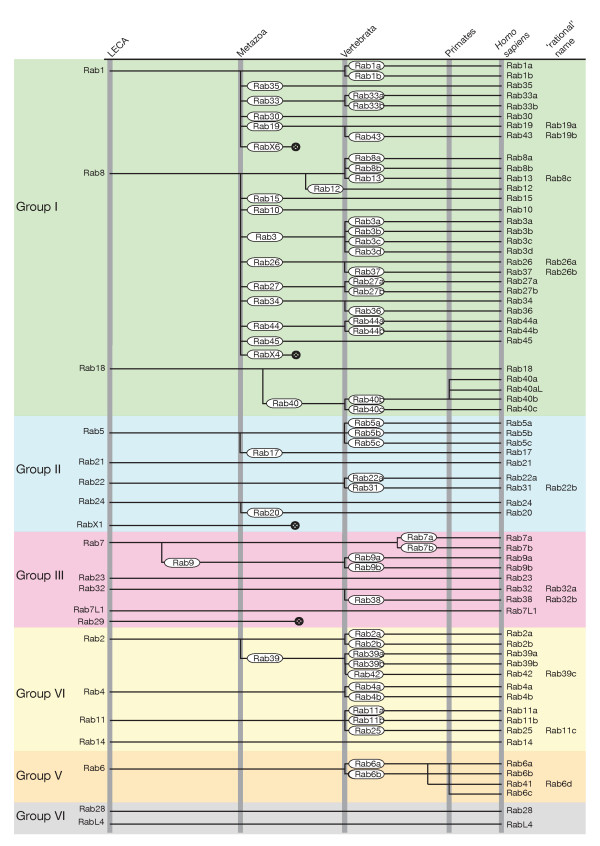
**Evolutionary history of the human Rabs**. Losses and gains of Rabs from the last eukaryotic common ancestor (LECA) towards *Homo sapiens*. The six supergroups are indicated by their name on the right and the common color scheme. The time points of major remodeling of the human Rab proteins are shown on the top. Most gains in the Rab repertoire are associated with the transition to multicellularity or the rise of vertebrates. In addition, there are two Rab proteins that are specific to primates (Rab40a, Rab40aL), Rab41 is present only in primates and dolphins, and Rab6c seems to be specific to *Hominidae*. The losses of RabX1, Rab29, RabX4, and RabX6 are indicated by their lines terminating somewhere between the development of multicellularity and the rise of the vertebrates.

The striking expansion of Rabs in humans seems to have been caused by two separate processes: one at the start of metazoan evolution and a second that occurred at the appearance of vertebrates. Comparing the Rabs of metazoans with those of choanoflagellates, which are the closest relatives of metazoans, the appearance of 17 new Rab proteins can be seen in metazoans (Figure 5). Interestingly, the large majority (14) of these new Rab proteins appeared in group I, indicating an early diversification of the exocytic pathway. It seems likely that this is associated with several changes in cellular organization. Not only are metazoans multicellular, but they also form polarized cell sheets, hence proteins need to be trafficked to different domains on the cell surface. In addition, even early metazoans seem to have had neuroendocrine cells that communicated with their neighbors, presumably by regulated release of compounds stored in vesicles [55]. These extra processes will have required diversification of the Rab machinery that is involved in delivery to the cell surface, and indeed, many of the relatives of Rab8 are believed to be involved in such processes. Several members of this family seem to be expressed primarily in the brain, and in some cases have been clearly shown to function in neurotransmitter release (Rab3, Rab15 and Rab27 and RabX4) [47]. It also seems possible that more complex patterns of exocytosis would have required a concomitant elaboration of pathways of endocytic recycling, which may account for the expansion of the Rab1 family to generate more Rabs associated with Golgi functions, such as Rab19/43, Rab30 and Rab33. By contrast, plants and fungi show no expansion of Rab8, suggesting that it was protein-mediated interactions between cells without cell walls, rather than multicellularity *per se*, which drove the Rab explosion in early metazoans.

The second major expansion in Rab numbers took place during the rise of the vertebrates, most likely through two rounds of whole genome duplications, expanding their set from 38 to 62 Rab proteins (with further duplications in primates, taking the total in humans to 66). In contrast to the first event, the effect of the expansion in vertebrates can be seen in all major groups, and indeed, many other vertebrate families show such an expansion from one gene to two, three or four paralogous genes (Figures 2; Figure 4). In the case of the Rabs these extra paralogs may have acquired subtly different roles, but in some cases, the paralogs are expressed in different sets of tissues, which may have contributed to their conservation [16,56].

Rationalizing Rab expansions in other kingdoms is made more difficult by the more restricted number of genomes available. However, it is at least clear that in plants several Rab families have also expanded, with the most notable being Rab11, which diversified even in Bryophyta. This also explains how the Angiospermae were able to encompass such a large variety of Rab11 proteins (we found 10 different proteins as a general pattern, and *A. thaliana *has 26 Rab11-related proteins). Rab11 is involved in recycling back to the plasma membrane, and diversification of such routes to the plasma membrane seem likely to be important for cell plate formation during mitosis, for polarized growth of root hairs and pollen tubes, and for the targeted delivery of auxin transporters that underlies the spatial organization of some multicellular structures in plants [57-59].

## Discussion

Using probabilistic models and advanced tree building methods, we have been able to build the most comprehensive evolutionary tree of the Rab family that has been reported to date. In our analysis, we defined the Rab family as being distinct from the Ran family, and thus excluded some proteins which have been referred to as Rab-like but which are more likely to be 'Ran-like' or have roles distinct from Rabs. These include RabL2, RabL3, and RabL5.

Our findings confirm and extend the evidence that the LECA had a large number of Rabs [16]. If our size estimate of LECA Rab repertoire is incorrect, it will, if anything, prove to be an underestimate once more genome sequences become available from non-metazoan organisms, in particular protozoa. Indeed after our analysis was completed, Elias *et al. *[60] reported an analysis of LECA Rabs using a different method to identify evolutionary relationships applied to only 55 species, and concluded that the LECA could have contained 23 Rabs. Although this 'ScrollSaw' method was not validated with well established protein families, and found only two of the six supergroups we have identified, there is nonetheless a reassuring overlap of 19 Rabs with our LECA repertoire (although Elias *et al *used non-standard names for Rab7L1 (Rab32B) and RabX1 (Rab50)). The one extra Rab in our proposed set is Rab29, which Elias *et al *did not distinguish from Rab7L1, possibly due to their use of a smaller genome set, but as discussed above, we felt we could tentatively include Rab7L1 in the LECA repertoire, based not only on it being in amoeboza and metazoans, but also on it being an excavate sequence that scores above the strict cut-off with our Rab29 HMM. Elias *et al. *also suggest that the LECA contained four further Rabs that we did not include; Rab20, Rab34 and RabL2 (which they renamed RTW), and a new family that they named RabTitan. They based Rab20 on four sequences outside of unikonts, but in each case the relevant sequence gave a higher score with our Rab24 model, and the organism concerned had no other Rab24 in its genome, suggesting that these really are Rab24s. The inclusion by Elias *et al. *of Rab34 is based on a Rab34-like sequence that is present in one non-unikont genome (the excavate *N. gruberi*). With our Rab34 HMM, this sequence gave a score below the strict cut-off point, and so we felt it premature to include this protein. We also did not include RTW/RabL2, as it is well established to be an outlier from the Rab family that is more closely related to Ran, and so is unlikely to be a Rab [14,15]. RabTitan is based on a set of rather distantly related sequences from several kingdoms, but all lack C-terminal cysteines and seem only very distantly related to the Rabs. We tested a 'RabTitan' from a metazoan (*Branchiostoma floridae*), an amoebozoan (*Dictyostelium discoideum*) and a excavate (*N. gruberi*), and in each case we obtained a much lower score for our general Rab HMM than for either Ran or Rho/Rac from the corresponding species. To determine from which G proteins these RabTitans evolved will probably require a phylogenetic analysis of the entire Ras superfamily.

Irrespective of whether any further Rabs will be added to the LECA repertoire, it now seems unambiguously established that the repertoire was large, probably consisting of at least 20 members, which strongly supports the notion that the LECA had a complex set of internal organelles and trafficking steps [16,61]. This is consistent with the 'complexity early' model of eukaryotic evolution that has been suggested by examining other families of proteins such as cytoskeletal components and motors [24,31,62]. The notion that a single-celled LECA needed such complexity in its internal membranes is consistent with the Rab repertoires of more than 50 members found in some extant single-cellular protozoa [63-65].

The widespread loss of Rabs during the diversification of eukaryotes probably reflects in part the loss of particular structures or processes during specialization. These include cilia/flagella and a capacity for phagocytosis. Interestingly, a LECA Rab of unknown function, Rab28, has a phylogenetic distribution similar to that of cilia, and it groups with RabL4, a Rab known to be involved in cilia function. Thus we suggest that Rab28 is a candidate for a role in cilia formation or function. In addition, the requirements for membrane-trafficking events during cytokinesis are likely to have changed as this process has diversified greatly in different lineages [66].

It is possible that some cases of Rab loss reflect overlap in function between particular Rabs that belong in the same family, even if they have diverged from a common ancestor in the LECA. For instance, Rab4 and Rab11 were both present in the LECA, but are part of the same fundamental group of Rabs and have been found to share some effectors [67]. This may have allowed Rab4 to have been lost more readily than if Rab11 had not been present to take over some of its roles. Likewise, mammalian Rab5 shares the same binding site on some effectors with other LECA Rabs of its group, Rab21 and Rab22, and again the latter have often been lost during evolution [68]. Such 'cryptic' redundancy suggests that caution may be needed in interpreting the effects of deleting particular Rabs; that is, a failure to see a perturbation of a particular process need not imply that the Rab that has been deleted is not normally involved in that process. This is more likely to be the case for Rabs that have duplicated more recently in evolution, and may explain the surprisingly mild phenotypes observed after loss of some mammalian Rabs such as Rab18 or the Rab3 family, as both have relatives that arose from duplications at the dawn of metazoan evolution [67,69].

Not only have some Rabs been lost, but also new ones have been gained by gene duplication and divergence. A large increase in the Rab repertoire occurred at the root of the metazoan lineage, which may be linked to multicellularity without a cell wall, and hence the opportunity for intracellular contacts and communications to be mediated by proteins directed to the cell surface. In most cases it seems likely that the duplications generated Rabs that carry out spatially or functionally related roles. However, there are one or two enigmatic exceptions; for instance, Rab35 is related to Rab1 and yet has a role in endocytosis at the plasma membrane rather than acting at the Golgi [70,71], while Rab2 emerged in a LECA group with Rab4, Rab11, and Rab14, and yet acts on the Golgi rather than on endosomes [72].

For the non-metazoan kingdoms, a lack of sufficient genome sequences restricts a deep understanding of Rab expansions, although plants seem to have expanded Rab11 [73]. The protozoans *Paramecium, Trichomonas, Entamoeba *and *Tetrahymena *have greatly expanded their Rab repertoires, perhaps reflecting the complex endocytic/phagocytic routes in these organisms and their elaborate cilia or flagella, but understanding the history of these expansions will require more genome sequences from other excavates and chromalveolates [63-65,74,75].

A second round of Rab expansion occurred in vertebrates as a result of the two rounds of genome duplication that seem to have happened early in vertebrate evolution and that also generated paralogs for many other gene families [76]. It remains to be determined which of these new Rabs have been conserved because they had different functions or different expression patterns [16,77]. However, it is clear that the naming of these paralogs has not been consistent, with some being distinguished with letters (for example, Rab4a and Rab4b) and others by new numbers. For instance, Rab25 could be Rab11C, and Rab34 and Rab36 could be Rab34a and Rab34b. Adopting a consistent nomenclature for the vertebrate Rabs might avoid some confusion, and in particular the risk that potentially highly redundant paralogs are overlooked because they have different numbers. These anomalies and our suggested 'rational' names for human Rabs are shown in Figure 5.

Finally, it is interesting to compare the evolutionary patterns of Rabs with those of other components of membrane traffic. Known vesicle coat proteins (adaptor proteins (AP)-1 to AP-4, retromer) and multi-subunit tethering complexes (conserved oligomeric Golgi (COG), Golgi-associated retrograde protein (GARP), exocyst, homotypic fusion and vacuole protein sorting (HOPS) and transport protein particle (TRAPP)) have undergone little expansion since the LECA, indicating that their different subtypes correspond to the fundamental routes of traffic [78-81]. These protein families must be the core components of ancient machineries involved in the fundamental steps of vesicle budding and fusion, which diversified during evolution of the LECA to adapt to the needs of specialized compartments. The SNARE protein families show a post-LECA diversification pattern comparable with that of Rabs; for example, during the rise of metazoans, the development of vertebrates [82], and in angiosperms [83]. In addition, metazoan SNARE diversification is, like Rab8 expansion, associated with those SNAREs involved in transport steps to the plasma membrane. However, the Rab proteins show a much more extensive and dynamic gain-and-loss history, with SNARE proteins having a tendency to become essential 'housekeeping' genes. For example, *S. cerevisiae *has retained only 6 of the 20 LECA Rabs but 21 of the 22 LECA SNAREs.

This greater diversity in Rab evolution may reflect the fact that it is difficult to duplicate a coat, a tethering complex, or a SNARE tetramer, as they are encoded by multiple genes. More importantly, it suggests that a particular Rab can be gained or lost without a concomitant gain or loss of any of the known members of the known coat, SNARE, or tethering complex families. This is rather unexpected, as it implies that either these components are associated only with those Rabs that are 'indispensible', or that the Rabs only add functionality to existing processes, or, as seems likely in at least some cases, new Rabs can add transport steps without the cell having to evolve new coats, tethering complexes, or SNAREs. This suggests that Rabs have directed the evolutionary plasticity of membrane traffic, and hence, by implication, they encode much of its specificity. Given that the function of many Rabs, including some of those that were present in the LECA, is poorly understood, it seems certain that there is still much to learn about the involvement of Rabs in the organization of internal organelles and trafficking pathways.

## Conclusions

In this study, we built an evolutionary tree of the Rab family that reconstructs the repertoire of Rabs in the LECA and accounts for the origins of all 66 Rabs in the human genome. The twenty different Rabs of the LECA seem to have arisen by duplication and diversification of six fundamental Rab types, which probably can be assigned to the fundamental routes of membrane traffic. Indeed, many extant eukaryotes have fewer Rabs than the LECA, although in some cases this has been balanced by expansion and diversification of the remaining Rab set. The metazoans seem to be the kingdom that has lost fewest of the LECA Rabs, although all individual phyla within metazoans have lost one or more of them. Patterns of Rab loss or expansion can in some cases be correlated with changes in membrane-traffic processes, and we found that a LECA Rab of unknown function, Rab28, showed a pattern of loss similar to that of cilia, suggesting a role for the protein. The evolutionary plasticity of Rabs has been much greater than that of other membrane-traffic components such as coats, tethers, and even SNAREs. This suggests that variation in Rabs has been used during evolution to augment membrane-traffic processes and even to invent new ones, and hence to generate the diversity of organelle function that exists between different cell types and organisms.

## Methods

### Sequence classification

We started with a set of approximately 500 Rab proteins from 21 species: *Arabidopsis thaliana, Aspergillus fumigatus, Aspergillus niger, Batrachytrium dendrobatidis, Caenorhabditis elegans, Chlamydomonas reinhardtii, Dictyostelium discoideum, Drosophila melanogaster, Entamoeba histolytica, Giardia lamblia, Homo sapiens, Laccaria bicolor, Monosiga brevicollis, Nematostella vectensis, Plasmodium falciparum, Saccharomyces cerevisiae, Schizosaccharomyces pombe, Tetrahymena thermophila, Trichoplax adherens, Trypanosoma brucei, and Volvox carteri*. We used two methods for our classification analysis. Firstly, we made use of the CLuster ANalysis of Sequences (CLANS) software, which uses the Basic Local Alignment Search Tool (BLAST) and a subsequent similarity analysis to identify similar subtypes [84]. Using the implemented network clustering method and different e-value cut-offs, we constructed a hierarchical representation of the analysis. Secondly, we used phylogenetic analysis. We aligned the sequences, extracted the 163-residue conserved Rab motif and reconstructed a phylogenetic tree as described below. The results of the analyses were inspected for subgroups which showed similar structure with both analyses and also contained a large number of species from different eukaryotic kingdoms. These were used to define an initial set of 15 subgroups of the Rab family. For each subgroup we used HMMER, with standard settings and calibration for the profiles, to build HMMs [85], and to then search the National Center for Biotechnology Information (NCBI) RefSeq database.

To ensure the quality of the HMMs we added a verification step after gathering new sequences. All sequences in our classification were inspected by eye. Our verification visualized the HMM hits for the different models, and allowed us to review the fit of the sequence to our classification. For the vast majority of the sequences we found a clear fit to one model; however, for a small number of sequences, this was not the case and we inspected these further. There may be several reasons why a sequence will not fit well within the classification. If a species has an eccentric evolutionary history (for example, *G. lamblia*) it can reduce the fit of the models. However, we found that most problems were due to low sequence quality, and this was especially common with Expressed Sequence Tag (EST) sequences. Our verification method allowed us to use pairwise alignments and BLAST searches to evaluate the general fit of the sequence. Identified fragmented sequences were joined into one sequence, and sequences that were obviously misassembled were removed from the dataset.

The verification was especially important because we integrated sequence information from a large range of different databases of differing quality. We generally ranked the reliability of the different databases in the following way. Sequences from the NCBI RefSeq database were considered to be most reliable, followed by NCBI Nr and NCBI EST, and then all other databases. For duplicate entries, we generally kept the sequence from the highest ranked database, and if there was more that one from this database we kept the latest version of the sequence. We should emphasize that at each iteration of the classification, we manually inspected all the sequences that showed a low fit to our model and also any new sequences. In the first iteration we gathered approximately 3000 sequences, and after the verification, we obtained a set of 1400 sequences. To refine our classification we used the same methods as described in the previous two paragraphs. We defined a set of 20 different **s**ubgroups, each subgroup representing a Rab present in the LECA. Because these subgroups have undergone extensive duplications within the metazoans we supplemented these 20 subgroups with 15 further subgroups representing those metazoan duplications. Because the resulting dataset showed a clear over-representation of metazoan sequences, we decided to construct our HMMs from a subset of all available sequence information.

For the 20 general models, we selected a set of 81 species that were more equally distributed across the eukaryotic domain. For the Rab9 model, we selected 94 Opisthokonta species, and for the metazoan models, we selected 53 species (see Additional file 3). Except for the Rab29 model, this general selection provided sufficient sequences; for Rab29, we randomly added sequences to reach 20 sequences. Finally, we supplemented the sequences from these genomes with additional sequences from the NCBI Nr and EST database whenever necessary. To further enrich our dataset, we obtained 43 genomes from the Broad Institute (http://www.broad.mit.edu), 86 genomes from the Department of Energy Joint Genome Institute (http://genome.jgi-psf.org), and integrated EST sequences from 236 species from the NCBI EST database. In total, we obtained more than 13,000 Rab sequences, of which we removed approximately 5,000, because they were duplicate entries, of low quality, or contained only a very short part of the Rab motif.

To resolve the five metazoan groups that we could not link unambiguously to one LECA Rab (Rab19, Rab33, Rab34, Rab44 and Rab45), we used a pBLAST-based similarity search [86]. For each group we blasted each of the sequences in the group against all of the Rab sequences from the same species. From the result, we took the sequence with the best expectation value as the closest relative. We counted the occurrences for the different LECA groups, and selected the group with the most similar sequences as its parent. Because each of the five groups has an independent HMM model for searches, the effect on the overall performance of the classification scheme appeared to be negligible; however, future research may be able to shed more light on these Rab proteins.

### Classification analysis

To estimate the quality of our classification, we used two different approaches. We estimated the positive predictive rate (PPR; a value that gives the rate at which a positive is a true positive) and the sensitivity (a value that describes the percentage of correctly found true positives) by resampling 90% of the members of each HMM 1000 times (see Additional file 2). To estimate the expectation value cut-offs for our models, (that is, the value at which we are confident that the Rab sequences are correct), we used approximately 2000 members of the larger Ras family to which Rab proteins belong (for example, Arf, Arl, Rho and Ran proteins). We provided two different cut-offs using the 5% and 95% percentiles as estimators. The first (strict) cut-off was generated using the 5% percentile of the expectation value distribution from the motifs we selected during our sequence analysis as member of the HMM., while the second (soft) cut-off was generated using the 95% percentile of the expectation value distribution from the motifs found by the HMM but belonging to the non-Rab sequences of the Ras family. The box plots of both samples together with the bounds for the models can be found in Additional file 2.

### Sequence alignment and phylogeny

Alignments were generated using the software Multiple Sequence Comparison by Log-Expectation (MUSCLE) [87]. To increase the quality of the alignments, we visually inspected the results and removed sequences of low quality, corrected the alignment when necessary, and removed sites with more than 25% gaps or sequences with more than 25% gap characters in the motif. The resulting alignments of groups I to VI are available as FASTA format files (see Additional file 4; Additional file 5; Additional file 6; Additional file 7; Additional file 8; Additional file 9). For phylogeny, we reconstructed a tree from the conserved alignments using Important Quartet Puzzling and Nearest Neighbor Interchange (IQPNNI), with a gamma distribution as a model for rate heterogeneity [88]. The estimation for the gamma distribution parameter used four rate categories. Additionally, the proportion of invariable sites was estimated from the data. We explored the use of the Jones, Taylor, and Thornton (JTT) and the Whelan and Goldman distance matrices for the reconstruction of our phylogenetic trees. Because we could not identify an advantage of one over the other, we selected the JTT for the analysis. The stopping rule of the algorithm was used, but the algorithm had to run for at least the suggested number of iterations. All other settings of the application were set to the default values. For each edge of the constructed tree, we estimated the confidence using likelihood mapping [89]. Secondly, we used the PHYLogeny Inference Package (PHYLIP) to apply a distance-based bootstrap analysis with 1000 replicates [90]. We used standard settings for 'seqboot', the JTT distance matrix once again and also a gamma distribution (with parameter approximation from TREE-PUZZLE [89] for 'protdist', and standard options for 'neighbor'). Whenever necessary, we used a random seed of nine. Because bootstrap values have been shown to be systematically biased, we used the almost unbiased (AU) test to correct for this [91]. The site wise log-likelihoods needed for the AU test were obtained using the Phylogenetic Estimation Using Maximum Likelihood (PhyML) software, and the test was performed using CONSEL [92,93]. We joined the results of both estimations, using the IQPNNI tree as a starting point, and labeled the inner edges of the tree with their likelihood-mapping and corrected-bootstrap support values. The resulting trees are available from TreeBASE (S12811), or in Nexus format from our *Rab *Database web server (see below).

### A web server for access to our results and the *de novo *classification of Rab proteins

We have implemented a web-based interface called *Rab *Database to provide access to our results (http://bioinformatics.mpibpc.mpg.de/rab/). It is divided into three sections. The first section provides access to our collected information, which can be searched for groups, species, and protein names. The second section allows submission of new sequences to our HMM models. We implemented the expectation value cut-off to reflect the strict and soft bounds for each family (defined in Additional file 2). The results display the best four hits and the position of the motif in the alignment. The final section contains the protein alignments and the Rab trees generated for this analysis in Nexus format, which can be analyzed in detail with SplitsTree [94,95].

## Competing interests

The authors declare that they have no competing interests.

## Authors' contributions

THK, DF, and SM designed the analysis, THK and NK performed the analysis, NK built the *Rab *Database web server, and SM and THK wrote the manuscript. All authors read an approved the final manuscript.

## Supplementary Material

Additional file 1 **Overview of Rabs in representative species outside of opisthokonts**. Species from various phyla are as indicated and where there is presence of at least one Rab of the given type, this is indicated by a cross. The species are from the four eukaryotic supergroups, S+C, SAR+CCTH; A, Archaeplastida; Ex, Excavata; Unik, Unikonta. Format: PDF. Size: 94 kb. Click here for file

Additional file 2 **Statistical validation of Rab classifications. (A) **To assess the quality of our hidden Markov models (HMMs), a resampling method was used, and 90% of the sequences used to generate each model were randomly gathered, and new models generated from these. The other Rabs were used as the search database, with a fixed size of the database of 100,000 sequences. The profile with the best expectation value was assumed to be the correct class, and the resampling was repeated 1000 times. For each model, the PPR (grey, left) and the sensitivity (black, right) are shown. All models achieved at least 95% PPR and sensitivity. False positives and false negatives occurring within the analysis were inspected and it was found that for a number of cases, the wrong classification was caused by metazoan-specific duplications. For example, Rab10 had the lowest observed sensitivity (95.4%), but on further investigation all false negatives were identified as Rab8 (42% of the false positives). Because Rab10 is a metazoan duplication of Rab8, the classification is not exactly wrong but is rather inaccurate. The metazoan models are likely to improve once further metazoan genomes become available. **(B-D) **As Rab proteins are members of the larger Ras protein family we needed to also address the problem of randomly identifying non-Rab sequences with the models. Because the models were generated using Rab sequences, non-Rab sequences should show a weaker fit to the model. To determine how specific the models were for Rab proteins, approximately 2000 members of the larger Ras family (for example, Arf, Arl, Rho and Ran proteins) were selected, and each of the models was used to predict 'Rab' motifs in these non-Rab sequences. We visualized the results using box plots with the 5% and 95% percentiles, shown as whiskers. Plots show the scores from sequences that were accepted to be members of the family modeled **(B)**, or from the set of non-Rab sequences that we selected **(D)**. For both graphs, the negative logarithm of the expectation value achieved by the motifs was plotted. The difference in e-value distribution between the two datasets was then used to define cut-offs for the confidence of our predictions. The first 'strict' cut-off was generated using the 5% percentile of the expectation value distribution in (B), and the second 'soft' cut-off was generated using the 95% percentile of the expectation value distribution from (D). Values are displayed in (C). Format: PDF Size: 1.6 mb. Click here for file

Additional file 3 **Overview of the species used to generate Rab-specific HMMs**. Species and species subsets used in computations and figures, showing species name, the abbreviation used for the species, the source database from which the sequences were obtained and the set in which it was used: models for the LECA groups, models for opisthokont groups, and models for metazoan-specific groups. Format: XLS Size: 29 kb. Click here for file

Additional file 4 **Alignment of group I Rab proteins in FASTA format**. Format: TXT Size: 147 kb. Click here for file

Additional file 5 **Alignment of group II Rab proteins in FASTA format**. Format: TXT Size: 70 kb. Click here for file

Additional file 6 **Alignment of group III Rab proteins in FASTA format**. Format: TXT Size: 53 kb. Click here for file

Additional file 7 **Alignment of group IV Rab proteins in FASTA format**. Format: TXT Size: 86 kb. Click here for file

Additional file 8 **Alignment of group V Rab proteins in FASTA format**. Format: TXT Size: 25 kb. Click here for file

Additional file 9 **Alignment of group VI Rab proteins in FASTA format**. Format: TXT Size: 16 kb. Click here for file
