# Supplementary material for: Untangling the evolution of Rab G proteins: implications of a comprehensive genomic analysis
Source: BMC Biol. 2012 Aug 8;10:71. doi: 10.1186/1741-7007-10-71 (PMC3425129; doi:10.1186/1741-7007-10-71)
Supplement: Additional file 1 — Overview of Rabs in representative species outside of opisthokonts. Species from various phyla are as indicated and where there is presence of at least one Rab of the given type, this is indicated by a cross. The species are from the four eukaryotic supergroups, S+C, SAR+CCTH; A, Archaeplastida; Ex, Excavata; Unik, Unikonta. Format: PDF. Size: 94 kb. [file 1741-7007-10-71-S1.PDF]

Additional file 1

			Group I			Group II					Group III					Group IV				Group V	Group VI	
	**Phylum**	**Species**	Rab1	Rab8	Rab18	Rab5	Rab21	Rab22	Rab24	RabX1	Rab7	Rab23	Rab29	Rab32	Rab7L1	Rab2	Rab4	Rab11	Rab14	Rab6	Rab28	RabL4
S+C	Ciliophora	*Paramecium tetraurelia*	x	x		x	x	x		x	x					x	x	x		x		x
	Ciliophora	*Tetrahymena thermophila*	x	x	x	x	x	x			x				x	x	x	x		x	x	x
	Rhizaria	*Bigelowiella natans*	x	x	x	x	x	x	x	x	x	x		x	x	x	x	x		x		x
	Cryptophyta	*Guillardia theta*	x	x	x	x	x	x		x	x			x		x		x	x	x	x	x
	Haptophyta	*Emiliana huxleyi*	x	x	x	x			x	x	x					x		x		x		x
A	Rhodophyta	*Cyanidioschyzon merolae*	x		x						x					x		x		x		
Ex	Percolozoa	*Naegleria gruberi*	x	x	x	x	x		x	x	x	x	x	x		x	x	x	x	x	x	x
	Metamonada	*Trichomonas vaginalis*	x	x	x	x	x	x		x	x	x		x	x	x	x	x	x	x		x
	Jakobida	*Reclinomonas americana*	x	x	x	x	x	x		x	x	x		x	x	x		x	x		x	x
	Metamonada	*Giardia intestinalis*	x											x		x		x		x	x	
Unik	Amoebozoa	*Dictyostelium discoideum*	x	x	x	x	x	x	x	x	x		x	x	x	x	x	x	x	x		
	Amoebozoa	*Dictyostelium fasciculatum*	x	x	x	x	x	x	x	x	x			x	x	x	x	x	x	x		
	Amoebozoa	*Dictyostelium purpureum*	x	x	x	x	x	x	x	x	x		x	x	x	x	x	x	x	x		
	Amoebozoa	*Polysphondylium pallidum*	x	x	x	x	x	x		x	x		x	x	x	x	x	x	x	x		
	Amoebozoa	*Entamoeba histolytica*	x	x	x	x	x	x			x			x	x	x		x		x		
	Amoebozoa	*Entamoeba dispar*	x	x	x	x	x	x			x				x	x		x		x		
	Apusozoa	*Thecamonas trahens*	x	x	x	x	x		x	x	x	x	x	x	x	x		x	x		x	
