# Supplementary figures and images for: Untangling the evolution of Rab G proteins: implications of a comprehensive genomic analysis
Source: BMC Biol. 2012 Aug 8;10:71. doi: 10.1186/1741-7007-10-71 (PMC3425129; doi:10.1186/1741-7007-10-71)

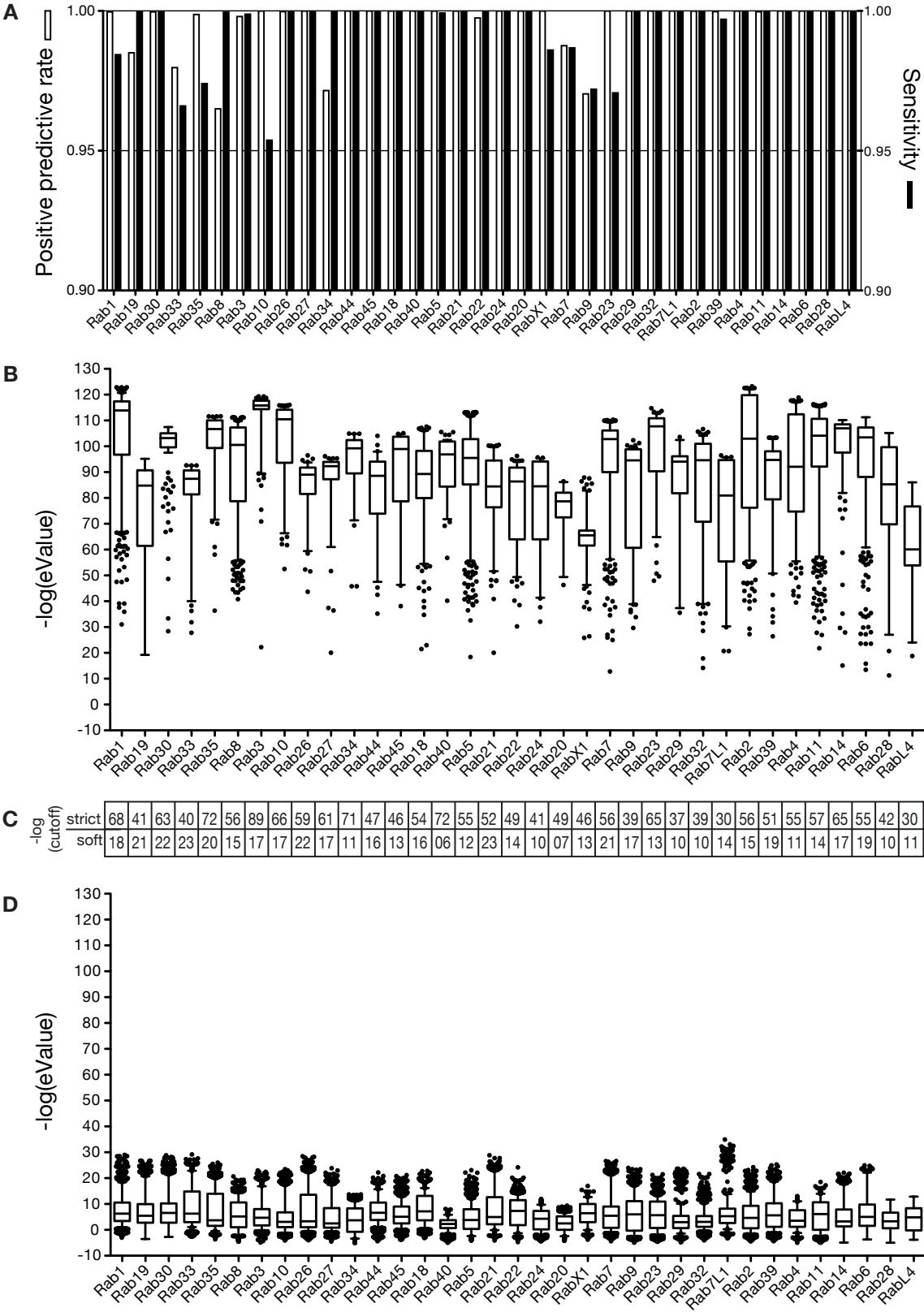

Supplement: Additional file 2 — Statistical validation of Rab classifications. (A) To assess the quality of our hidden Markov models (HMMs), a resampling method was used, and 90% of the sequences used to generate each model were randomly gathered, and new models generated from these. The other Rabs were used as the search database, with a fixed size of the database of 100,000 sequences. The profile with the best expectation value was assumed to be the correct class, and the resampling was repeated 1000 times. For each model, the PPR (grey, left) and the sensitivity (black, right) are shown. All models achieved at least 95% PPR and sensitivity. False positives and false negatives occurring within the analysis were inspected and it was found that for a number of cases, the wrong classification was caused by metazoan-specific duplications. For example, Rab10 had the lowest observed sensitivity (95.4%), but on further investigation all false negatives were identified as Rab8 (42% of the false positives). Because Rab10 is a metazoan duplication of Rab8, the classification is not exactly wrong but is rather inaccurate. The metazoan models are likely to improve once further metazoan genomes become available. (B-D) As Rab proteins are members of the larger Ras protein family we needed to also address the problem of randomly identifying non-Rab sequences with the models. Because the models were generated using Rab sequences, non-Rab sequences should show a weaker fit to the model. To determine how specific the models were for Rab proteins, approximately 2000 members of the larger Ras family (for example, Arf, Arl, Rho and Ran proteins) were selected, and each of the models was used to predict 'Rab' motifs in these non-Rab sequences. We visualized the results using box plots with the 5% and 95% percentiles, shown as whiskers. Plots show the scores from sequences that were accepted to be members of the family modeled (B), or from the set of non-Rab sequences that we selected (D). For both graphs, the negativ [file 1741-7007-10-71-S2.PDF]
